# Editorial: Oncogenic PI3KT/Akt/mTOR pathway alterations, ROS homeostasis, targeted cancer therapy and drug resistance

**DOI:** 10.3389/fonc.2024.1372376

**Published:** 2024-02-16

**Authors:** Rozangela C. Pedrosa, Karina B. Felipe, Danilo Wilhelm Filho

**Affiliations:** ^1^ Laboaratory of Experimental Biochemistry, Department of Ecology and Zoology, Federal University of Santa Catarina, Florianopolis, Santa Catarina, Brazil; ^2^ Laboratory of Physiology and Cell Signaling, Department of Clinical Analyses, Federal University of Parana, Curitiba, Parana, Brazil

**Keywords:** cancer, PI3KT/Akt/mTOR pathway, cancer therapy, drug resistance, apoptosis

The present Research Topic include several distinct articles that address the exploration of PI3KT/Akt/mTOR pathway, alterations, and their inhibitors both synthetic and derived from natural sources—as well as the investigation of immune and nutritional checkpoint inhibitors through diverse strategies which holds the potential to unveil small molecule inhibitors characterized by high selectivity and low toxicity in the realm of anti-cancer drug development.

Despite the approval of several drugs for breast cancer treatment, such as Alpelisib, therapeutic options targeting the PIK3CA gene mutation are limited, and several reports revealed higher rates of adverse events among patients, including diarrhea, appetite loss, nausea or vomiting, and fatigue. Since its approval there have been compelling arguments advocating for the withdrawal of the drug from the market due to concerns about its tolerability and effectiveness in real-world clinical practice, as recommended by Alaklabi et al. They emphasize the necessity of comprehensive real-world assessments to better inform clinical decision-making and enhance patient care outcomes.

Some investigations have revealed that diminished Harakiri (HRK) gene expression contributes to the initiation and progression of cancer. In colorectal, gastric, and prostate cancer, HRK expression is frequently low or absent. Wang et al. demonstrated experimentally that HRK exhibits low expression levels in colorectal cancer tissues. Notably, this study provided evidence that HRK promotes apoptosis and inhibits the proliferation of colorectal cancer cells by suppressing the PI3K/AKT/mTOR signaling pathway. Consequently, HRK emerges as a potential therapeutic target for the treatment of colorectal cancer.

*Asparagus officinalis* extracts and their compounds from Traditional Chinese Medicine exhibit noteworthy activities against diverse types of cancer cells, including the inhibition of cell proliferation, induction of apoptosis/cell-cycle arrest, as well as suppression of invasion through intricate signaling pathways. Li et al. used *in silico* tools incorporating network pharmacology, multidirectional pharmacology, and molecular docking to elucidate the mechanisms underlying the application of asparagus in treating multiple myeloma (MM). It also showed, among other results, that asparagus induced retardation and apoptosis of MM cells in the G0/G1 phase.

The cutaneous squamous cell carcinoma (cSCC) is a globally prevalent malignant cutaneous tumor, while 5-aminolevulinic acid photodynamic therapy (ALA-PDT) is a crucial therapeutic approach extensively employed in cutaneous cancer treatment. Nevertheless, clinical observations revealed substantial pain associated with conventional ALA-PDT, prompting patients to discontinue treatment. To address this Research Topic, previous investigations by Zeng et al. modified conventional ALA-PDT by reducing ALA incubation time and increasing light dosage (Modified Photodynamic Therapy, M-PDT), resulting in diminished pain and improved patient treatment adherence. The authors propose that the therapy disrupts autophagic flux, culminating in autophagosome-dependent apoptosis in cSCC. M-PDT induces cell death in cSCC through autophagosomes, mediated by the reactive oxygen species (ROS)-induced inhibition of the Akt/mTOR signaling pathway.

Ferroptosis represents a novel regulatory cell death mechanism intrinsically linked to redox reactions (mainly Fenton reaction in which transition metal ions, notably ferrous ions reacting with H2O2 forming hydroxyl radicals) able to enhance oxidative stress thereby killing cancer cells. Mounting evidence underscores the therapeutic potential of inducing ferroptosis in cancer cells, particularly when integrated with conventional therapies. Lu et al. comprehensively reviewed the signaling pathways governing ferroptosis, including XC−/GSH/GPx4, NAD(P)H-FSP1-CoQ10, GCH1-BH4-phospholipid, ATM-MTF1-Ferritin/FPN1, NRf2, and PI3K-AKT-mTOR. The authors highlight the substantial promise of ferroptosis in synergy with radiotherapy (RT) for cancer treatment, emphasizing its unique therapeutic effects, such as synergism, sensitization, and reversal of drug resistance, thereby paving the way for innovative cancer therapies. The activation of the PI3K-AKT-mTOR pathway hinders ferroptosis through the SREBP1/SCD1-mediated lipogenesis pathway. This mechanistic insight sheds light on the intricate interplay between the PI3K-AKT-mTOR axis and ferroptosis, presenting potential avenues for therapeutic intervention in cancer treatment.

The PI3K/Akt signaling pathway is intricately interconnected with Fatty Acid-Binding Proteins (FABPs), a family of intracellular lipid chaperones that serve as crucial regulators of fatty acid metabolism. Multiple studies across diverse cancer types consistently reveal that FABP5 exerts an anti-apoptotic influence, promoting cancer cell proliferation, colony formation, epithelial–mesenchymal transition (EMT), migration, invasion, and angiogenesis *in vitro*. Chen et al. assessed the impact of the FABP5/PPARγ and CaMKII signaling pathway on chemoresistance to doxorubicin in the BRCA cell line. The findings indicated that FABP5 enhances breast cancer resistance to doxorubicin through both the PPARγ and calcium signaling pathways. The insights provided by the FABP5/PPARγ and FABP5/CaMKII axes shed light on the mechanisms underlying doxorubicin treatment resistance in breast cancer.

Recent studies have associated receptor-interacting serine/threonine-protein kinase-2 (RIPK2) with tumorigenesis and malignant progression. Silencing RIPK2 has been found to decrease apoptosis, proinflammatory factors, and oxidative stress by activating the AKT pathway and suppressing NF-κB signals. In a concise review, You et al. evaluated the feasibility of targeting RIPK2 as an anti-tumor drug and summarized the research progress of RIPK2 inhibitors, demonstrating that inhibiting RIPK2 is deemed more destructive to tumors.

Tyrosine kinase inhibitors (TKIs), such as Dasatinib, Imatinib, Ponatinib, and Ruxolitinib, are commonly incorporated into the chemotherapy regimens for various leukemias. Nevertheless, the leukemia microenvironment’s growth factors and nutrients can sustain cell cycle and survival, even under TKI treatment targeting the driving oncogene. These stimuli converge on the mTOR kinase, with elevated activity associated with poor prognosis. In preclinical models of Ph+ and Ph-like B-ALL, mTOR inhibitors significantly enhance the anti-leukemic efficacy of TKIs. Despite the compelling rationale for mTOR targeting in B-ALL, the first two generations of clinically tested mTOR inhibitors, including rapalogs and mTOR kinase inhibitors, have not demonstrated a distinct therapeutic advantage. Addressing this limitation, Buono et al. conducted a review introducing novel therapeutic strategies for managing Ph-like B-ALL. They discuss innovative approaches to target mTOR in B-ALL, aiming to overcome the shortcomings of previous mTOR inhibitor classes. The review particularly highlights third-generation bi-steric inhibitors, selectively targeting mTOR complex-1 (mTORC1), and exhibiting preclinical efficacy with intermittent dosing. An important non-pharmacological strategy involves nutrient restriction to target signaling and metabolic dependencies in malignant B-ALL cells. Mechanistically, nutrient restriction inhibits the intracellular nutrient sensor mTORC1, concurrently activating AMPK to enforce catabolic reactions, leading to adaptive cellular stress responses. Nutrient restriction suppresses other signals such as PI3K/AKT and RAF/MEK/ERK (MAPK) pathways that collaborate with mTORC1 in promoting oncogenesis. Consequently, these cellular effects of nutrient restriction limit malignant cells’ ability to thrive and proliferate. Aligning with the authors’ proposed approaches, bi-steric mTORC1 inhibitors and nutritional interventions have the potential to potentiate TKI efficacy in Ph-like leukemia.

However, due to the extensive number, heterogeneity, and varying quality of publications related to mTOR and breast cancer, elucidating the current state of research in this domain is imperative for both, researchers, and clinicians. To address this need, Zhang et al. employed scientometric techniques and visualization tools to analyze a substantial volume of bibliographic metadata associated with the research area of mTOR and breast cancer utilizing Web of Science. The analyzed publications underscore the crucial involvement of mTOR in breast cancer pathogenesis. However, the precise mechanism of its action remains controversial, warranting further investigation. The scientometric analysis provides a comprehensive overview of the current state of research, shedding light on topical issues that merit further exploration in the realm of mTOR and breast cancer.

## Author contributions

RCP: Writing – original draft, Writing – review & editing. KBF: Writing – original draft, Writing – review & editing. DWF: Writing – original draft, Writing – review & editing.

